# Acceptance of Smart Contracts in Patients Receiving Primary Care: Exploratory Study

**DOI:** 10.2196/82237

**Published:** 2026-03-12

**Authors:** Mohamed Abdelhamid, Pamella Howell, Deepti Singh

**Affiliations:** 1 Department of Information Systems, College of Business, California State University Long Beach, 1250 Bellflower Boulevard, Long Beach, CA, 90840, United States, 1 562 985 2361; 2 Department of Information Systems, College of Business and Economics, California State University Los Angeles, Los Angeles, CA, United States

**Keywords:** blockchain, smart contracts, trust, perceived risk, perceived security

## Abstract

**Background:**

Health care has seen several new disruptive technologies. One such innovation is the introduction of blockchain smart contracts. These smart contracts are activated automatically once preprogrammed conditions are met. Smart contracts have improved patient outcomes, the efficiency of care delivery, and reduced costs. Despite their benefits, patients have had limited interactions with smart contracts in primary care; therefore, they may not trust blockchain-based smart contracts and may perceive them as risky or have concerns about their security.

**Objective:**

This study aimed to evaluate how patients’ perceptions of smart contracts affect their adoption in primary care. Specifically, we investigated the impact of patients’ perceptions of smart contract security, risk, and trust in their health care providers.

**Methods:**

This study used an experimental survey design to evaluate acceptance of smart contracts. Patients were randomly assigned to 1 of 2 research scenarios proposing either the positive use of blockchain smart contracts or the loss of benefits if a patient opted out. We collected data from a total of 387 participants. The Likert survey used 3 items to measure 5 constructs in the conceptual model. The 5 hypotheses were that gain-loss-framed messaging, perceived security, and trust would have a positive impact on the adoption of smart contracts, whereas perceived risk and the clinical setting would have a negative impact on patients’ intention to adopt smart contracts. The conceptual model was tested using structural equation modeling, and the model fit indices suggested a good fit.

**Results:**

Most of the hypotheses were supported, except for the gain-loss-framing effect. As hypothesized, perceived security had the strongest positive influence on the intention to use smart contracts (*β*=.50; *P*<.001). Trust in health care providers also showed a significant positive relationship (*β*=.43; *P*<.001), while perceived risk had a smaller but still significant negative impact (*β*=–.071; *P*.048). Patients in clinic-based settings had a lower intention to use smart contracts than patients in telehealth settings, and male patients had a lower intention to use smart contracts than female patients.

**Conclusions:**

The results of this study have implications for health care providers who intend to adopt smart contracts early, that is, early majority or late adopters. To facilitate their implementation, providers should highlight the security benefits of smart contracts and leverage patient trust. Providers should customize smart contract implementation strategies based on patient demographics such as age, health status, and gender. By understanding these factors, health care organizations can more effectively promote the adoption of smart contracts and realize the potential benefits of this disruptive technology in primary care.

## Introduction

### Background

Disruptive health care technology replaces existing technology; it benefits providers and patients because it reduces costs, promotes high-quality care, and is convenient [1,2]. Disruptive health innovation can affect processes, techniques, digital health education, diagnostics, device development, and the discovery of biological mechanisms underlying health and disease [3]. In this study, we proposed that smart contracts, a specialized blockchain mechanism, are a process-oriented disruptive health care technology that can successfully replace traditional business processes to facilitate care delivery.

The term “smart contract” refers to a computer program that runs on multiple distributed ledger nodes when predetermined conditions are met, without third-party intervention [4]. Essentially, smart contracts contain all of the details required to execute an agreement or workflow, and they run automatically when the conditions are met, without intermediaries. In health care, smart contracts have been applied to various use cases, such as remote patient monitoring [5], clinical services such as telesurgery [6], the internet of medical things [7], care coordination [8], facilitating research [9], and optimizing supply chains [10]. The adoption of smart contracts has improved patient outcomes and increased efficiency by enhancing the management of medical sensors in telehealth, improving care delivery, providing remote access to quality health care professionals, and reducing travel and care delivery costs. However, patients have not directly interacted with smart contracts. Therefore, it is necessary to understand patients’ feelings about the implementation of smart contracts that would directly affect their health care.

In a value-based payment model, providers hold specialized contracts with insurers. They are compensated for improved patient outcomes and for reducing unnecessary medical expenses rather than based on the number of patients they diagnose and treat [11,12]. Clinical quality initiatives, such as value-based care, are designed to reduce costs, enhance patient convenience, and improve the quality of patient outcomes. Unfortunately, smart contracts are in the early adoption phase in secondary and tertiary health care settings; therefore, only a few patients and providers have been introduced to or had personal experiences with the technology. In the primary care setting, smart contracts can replace patient and clinical workflows; however, many of the benefits will be unseen or behind the scenes. Although these smart contracts work seamlessly in the background, patients must first opt to participate in their use for care delivery; this is similar to the opt-in process used for health information exchanges. The need for patient buy-in underscores the importance of understanding patient perceptions. This study focused on the implementation of smart contracts in primary care and how patients’ perceptions may impact their further adoption.

Smart contracts can improve and standardize many workflows in the primary care setting; however, an equitable consideration of associated concerns is warranted. Patients may hesitate to adopt smart contracts due to trust issues or concerns about the risk and security of smart contracts. Trust is a complex construct in health care because patients may trust their physician but not necessarily the medical technology used in care delivery [13]. Individuals may perceive potential negative consequences, such as risks to their safety or personal data. Therefore, risk-averse individuals may be less inclined to use smart contracts to manage their health care. Modern smart contract stacks commonly support upgradeability, emergency pause mechanisms, and governance-controlled parameter changes. Although these patterns improve responsiveness, they introduce new security risks and concerns. Furthermore, the decentralized nature of blockchain-based smart contracts may raise privacy concerns regarding access to sensitive patient health information, because in a trustless environment, unknown third-party companies may facilitate smart contracts. These trust and privacy concerns are valid, as Ethereum, one of the third-party intermediaries proposed for implementing smart contracts in health care [14], experienced a security breach after unknown actors exploited a vulnerability in a decentralized autonomous organization (DAO). Hackers stole US $60 million from Ethereum’s DAO [15]. This security breach underscores the importance of examining sociotechnical issues that arise during the implementation of disruptive technology. The health care community must thoroughly understand the security, governance, and policies of blockchain technology [16] before mainstream adoption of smart contracts. With data breaches and privacy concerns receiving greater public attention, primary care patients are more informed, reinforcing the need to understand patient perceptions as smart contracts remain in early implementation.

This study is timely and addresses a gap in the literature because applications of smart contracts in health care have primarily focused on business-to-business or provider-to-provider interactions, inpatient care, and long-term care settings. Specifically, research has centered on the technical and organizational perspectives regarding adoption barriers in health care [17,18]; the patient perspective remains largely unexplored. Consequently, little is known about primary care patients’ willingness to authorize the use of smart contracts for their treatment or diagnosis. Therefore, this study used an experimental survey design to evaluate acceptance of smart contracts. To understand patients’ adoption of smart contracts in primary care, we examined key factors, such as patients’ trust in their health care providers, their perceptions of the risks involved in smart contracts, and their perceived security concerns.

Patients can benefit from innovations such as blockchain smart contracts. Therefore, examining the factors that influence patients’ willingness to adopt smart contracts is crucial, and herein lies our contribution to the literature. The conceptual model developed for this study can provide health care professionals with insights to expand the adoption of smart contracts and improve health care quality across the United States.

### Literature Review

The prior literature has established the unique attributes of blockchain technology, including decentralized consensus, predefined rules, accurate record-keeping, immutability, and a no-trust framework [19]. These features have proven useful in many industries, including health care. With the evolution of blockchain technology, smart contracts originated as self-executing contracts, with the terms of the agreement coded and shared between all parties, with predetermined conditions controlling further execution and enforcement [20]. In particular, blockchain technology shows great promise in health care because it offers solutions for data management, privacy, and security [20]. Moreover, blockchain technology can facilitate patient-driven interoperability in health care. Prior work has identified 5 key mechanisms: digital access rules, data aggregation, data liquidity, patient identity, and data immutability, while also addressing challenges such as transaction volume, privacy, and patient engagement [21]. Blockchain’s role has been examined in terms of exchanging data securely among health care professionals [22], providing better data accessibility in Electronic health record (EHR) platforms and smart contracts [23], low-latency health care apps [24], internet of things health care applications [25], and federated learning for at-home health monitoring [26].

Permissionless blockchains are decentralized systems that execute transactional programs and access a fully replicated, immutable data store without requiring complete trust in any single participant or third party. These systems are open to participation; anyone can join by running a local instance of the peer-to-peer protocol. Permissioned blockchains limit participation to a designated set of users for some or all roles within the system. This type of blockchain is particularly well suited for competing enterprises seeking to collaborate without relying on third-party networks [27,28]. There is a debate about which type of blockchain to use. Researchers argue that a permissioned network loses the benefit, since chaining transactions in a closed network is no longer able to protect the data from tampering [29]. Others argue that permissionless blockchains are susceptible to Sybil attacks, in which a hacker uses multiple identities to gain access to the blockchain [30,31]. Some researchers have suggested using smart contracts to switch between permissioned and permissionless blockchains [27]. Ultimately, the choice of which to use in health care will depend on the cost of implementation and the ability of the smart contracts to uphold laws such as Health Insurance Portability and Accountability Act (HIPAA) or General Data Protection Regulation (GDPR). In health care use cases, researchers have focused on permissioned private blockchains. If permissioned systems are to be implemented mainstream, providers may face a similar dilemma to health information exchanges in terms of implementation, such as governance [32], system architecture and standards, document and data items, consent, and usability [33,34]. An in-depth discussion of the challenges is outside the scope of this study; however, additional research is required to fully understand the implications and impact of implementation on interoperability and cost.

While the choice between permissioned and permissionless systems remains under consideration, the core attributes of blockchain technology hold considerable promise for health care transformation. The decentralized nature of blockchain technology provides enhanced security, privacy, efficiency, transparency, and trust, which highlights the potential of blockchain to revolutionize health care operations [35]. Smart contracts leverage blockchain’s secure and transparent nature to automate and enforce agreements without the need for intermediaries. Blockchain technology facilitates secure, private transactions and provides a transparent, immutable record, which enhances trust in smart contract applications [36]. Similarly, blockchain’s ability to improve data security and interoperability is crucial for the effective implementation of smart contracts between individuals and health insurance organizations [37].

Smart contracts thus offer significant potential for automating processes, reducing overhead, and improving efficiency in various sectors, including health care. They can also enhance data transparency in clinical trials by ensuring the integrity and immutability of trial data [38]. SmartAccess uses blockchain and smart contracts to enable transparent, auditable, and distributed access control for cross-organization medical data sharing [39].

One stream of smart contracts research is focused on the development of syntactic assets, algorithmic stablecoins, and the tokenization of the investment portfolio management process [40-42]. Meanwhile, another stream of research explores the use of smart contracts to solve key challenges in health care ecosystems, such as interoperability, automatic patient acquisition across multiple sites, and patient-centric identification and authentication [25,43,44]. Smart contracts are also being used to improve efficiency by automating processes and reducing the need for intermediaries [45]. While there are research streams exploring technical and operational challenges in the field of smart contracts in health care, patient perceptions of and concerns related to the smart contracts technology within the primary care framework in the United States have been largely unexplored.

Smart contracts have many applications in the health care industry. A systematic review of the role of smart contracts in creating sustainable health care platforms recognized key themes, such as health care process enhancement and patient privacy protection [46]. The impact of smart contracts has been realized in patient data management, medication adherence, insurance claims, clinical trials, supply chain management, network security, payment transfers, reducing medicine shortages, and many more applications. For instance, Fast Healthcare Interoperability Resources (FHIR) is a standard for exchanging health care information electronically [47]. Patientory is a blockchain-based platform that allows patients to store and share their health information securely [48]. Encrypgen uses blockchain and smart contracts for DNA data transfer to empower patients to make decisions regarding the sharing of their genomic data [49]. Smart contracts enable the secure and decentralized management of patient data, thereby ensuring data integrity and privacy [46]. They can also monitor patients’ adherence to their medication schedules and automate the insurance claim process to reduce administrative overhead and fraud [50]. Our focus on smart contracts in primary care has been motivated by the following properties, which are clearly distinct from EHR digitization:

1. Programmable enforceability: business rules such as referral eligibility, prior authorization criteria, and copayment logic are executable rather than merely documented; therefore, compliance is verifiable ex post via on-chain events.
2. Tamper-evident audit: an append-only log across organizations reduces disputes and supports audits.
3. Decentralized coordination: multiple parties including primary care providers (PCP), laboratories, and payers can share state without a single operator, thereby reducing reconciliation overhead.
4. Composability: smart contracts interoperate, enabling automated care pathways, for example, referral workflows that automatically trigger claims logic.
5. Governable change management: upgrades and pauses occur under explicit policy, with historical records preserved.

Interestingly, smart contracts present challenges in health care. Trust plays a crucial role in mediating the effects of the perceived usefulness and ease of use of smart contracts, as well as privacy concerns [51]. Privacy considerations are particularly important, with different applications of smart contracts (eg, immune certificates vs EHRs) affecting privacy evaluations differently. Technical, legal, and social factors also affect the adoption of smart contracts. Human factors, such as concerns about invasiveness and potential job displacement, can hinder the acceptance of smart health care systems [52].

Although resistance to change is a significant barrier to the acceptance of smart health care technology, it can be assuaged with careful management of the patient-provider relationship [53]. Trust in the patient-provider relationship, the reliability of the communication channel, altruistic motivation to contribute to health service quality for others, and the risk of losing information agency have been identified as determinants in patients’ adoption of smart health care technologies in general [54]. Yet, despite their potential benefits in addressing security, privacy, and data-sharing challenges [55], smart contracts face adoption barriers. However, demographic factors, including gender, age, and usage experience, can moderate the relationships between these factors and acceptance [51].

The scalability of blockchain networks can be another factor that limits the widespread adoption of smart contracts in health care. Ensuring that smart contracts comply with health care regulations and standards is therefore crucial. The implementation and maintenance of smart contracts require specialized knowledge and expertise. At the same time, the successful implementation of smart contracts in health care depends on patients’ willingness to accept and use this technology. Ensuring that new technologies align with patients’ needs and preferences is essential for providing high-quality, patient-centered care.

Overall, prior studies map a wide landscape of smart contract applications but provide limited critical synthesis across three decision-critical dimensions: (1) process locus (back-office claims vs point-of-care workflows such as referrals and prior authorization), (2) coordination model (single-operator platforms vs multiparty smart contracts that share state across organizations), and (3) assurance mechanism (narrative “security by design” vs engineering evidence such as audited libraries, governance controls, and formal methods). When organized along these dimensions, much of the literature emphasizes technical feasibility, cost, or compliance benefits. Very little empirical work examines patient intentions in multiparty smart contract settings that automate routine primary care tasks. This synthesis surfaces a specific research gap: a lack of empirical evidence on patients’ intentions to use smart contract–mediated workflows in primary care, where day-to-day activities (eg, referrals, prior authorization, and copayment settlement) depend on decentralized coordination and explicit assurance mechanisms.

Our study addresses this gap by estimating how perceived security, trust in provider stewardship and governance, perceived risk, and message framing are associated with intention to use primary care smart contracts whose mechanisms include programmable enforceability, tamper-evident audit trails, and governed upgrades.

### Hypotheses Development

#### Message Framing

Message framing, which involves presenting information in either a positive (gain-framed) or negative (loss-framed) manner, can significantly influence patient acceptance of smart contracts in health care. Research on health communication has shown that the way information is framed can affect attitudes, intentions, and behaviors related to health decisions. Gain-framed messages, which emphasize the benefits of engaging in a particular behavior, can be more persuasive than loss-framed messages, which highlight the consequences of not engaging in that behavior. Gain-framed messages are often more effective at promoting preventive health behaviors, such as using sunscreen or getting vaccinated [56].

Moreover, gain-framed messages can be more motivating because they focus on positive outcomes, which can be more appealing and less threatening to individuals. This aligns with the principles of prospect theory, which suggests that people are generally more motivated by potential gains than by potential losses [57]. The effectiveness of gain-framed messages can also depend on the characteristics of the target audience. For instance, individuals with a low preference for risk and a heuristic processing style are more likely to respond positively to gain-framed messages [58].

The framing of messages about blockchain technology and smart contracts can significantly influence patients’ perceptions and acceptance of their use in health care. Gain-framed messages that highlight how blockchain enhances the security and privacy of patient data could reassure patients. For example, emphasizing that blockchain ensures data integrity and gives patients control over their health information can make them feel more secure and in control [59]. By focusing on the positive outcomes, such as improved accuracy in medical records, faster access to patient history, and better coordination between health care providers, gain-framed messages can make patients more receptive to smart contracts in health care [60]. Highlighting the transparency and trust that blockchain brings to health care processes can also positively influence patient perceptions. For instance, explaining how blockchain can prevent fraud and errors in medical billing and insurance claims can build patient trust in the system [61].

In this study, a “smart contract” refers to a program that automatically enforces referral and prior authorization rules, logs each decision to a tamper-evident, cross-organization audit trail, and settles copayments when predefined conditions are met, without a single operator controlling the workflow. The framing manipulation, therefore, targets smart contract–specific attributes such as programmable enforceability, tamper-evident audit, and decentralized governance within primary care tasks such as check-in, referrals, prior authorization, and payment. These attributes are not generic benefits of digitization. Therefore, based on prior research, we hypothesized the following.

Hypothesis 1: In primary care smart contract workflows, a gain-framed description is associated with higher patient intention to use, relative to an otherwise identical loss-framed description.

#### Perceived Security

Perceived security refers to the degree to which individuals believe that their personal information is protected, safely managed, and secure when using blockchain smart contracts. In health care, perceived security is crucial due to the sensitive nature of patient information. A comprehensive review highlighted that security and privacy are paramount concerns in health care because breaches can lead to significant harm [62]. The study emphasized that addressing security concerns can enhance trust and the adoption of new technologies. Factors such as employees’ training on security issues, monitoring, ethics, and technical protection contribute to this perception. When patients feel their data are secure, their trust in the health care system increases. In health care, the use of smart contracts could lead to higher adoption rates due to the perceived security they offer. Smart contracts could streamline the management of patient data, thereby ensuring that only authorized personnel have access. This would not only improve data security but also enhance patient privacy, thereby potentially increasing patients’ intention to use such technology. Research on the telemonitoring of chronic conditions has shown that a sense of security is vital for patient safety and engagement [63].

The HIPAA regulates security among patients and health care providers. Smart contracts can automate administrative processes to ensure data integrity and security for the purpose of regulatory compliance. In addition to regulatory compliance, the underlying mechanisms in smart contracts use mathematical models as best practices to enhance security and trust through a rigorous verification process, whereby smart contracts encode legal or business terms using a translation process to verify that requirements are met [64]. Smart contracts can help health care organizations validate correctness, especially in settings where errors can affect patient safety. If patients are aware that smart contracts can improve precision in care delivery, their perceived security may increase, thereby enhancing their intention to use smart contracts for care delivery.

In addition, the quality of service (QoS) in health care applications encompasses aspects such as availability, reliability, performance, and security. Enhancing QoS can lead to increased patient trust and perceived security, which can lead to the increased adoption of smart contracts. A novel approach to improve the QoS of smart contracts in health care demonstrated that improved QoS can enhance patients’ perceived sense of security, reduce costs, and increase intention to use [6].

In our setting, smart contracts would automate prior authorization, referral routing, and copayment settlement between the PCP, laboratories, and payers. The relevant security property is a tamper-evident, append-only audit trail for these multiparty actions, with cryptographic access control to protected health information pointers (on-chain events and off-chain records). Thus, the perceived security construct is defined as confidence in these ledgered, cross-institution workflows rather than in health IT in general. This leads to our second hypothesis.

Hypothesis 2: Higher perceived security of tamper-evident, cross-organization audit trails and cryptographic access control is associated with a higher intention to use PCP-orchestrated smart contracts.

#### Trust in Health Care Providers

Trust in health care providers is a fundamental aspect of the patient-provider relationship and significantly influences patients’ willingness to share sensitive information and adhere to medical advice. This trust extends to the acceptance of new technologies recommended by health care providers, including smart contracts. When patients trust their health care providers, they are more likely to follow medical advice and accept new technologies recommended by them. Research has emphasized that trust in physicians is built through competence, effective communication, and professionalism [65]. This trust can extend to the technologies that physicians endorse, including smart contracts. For instance, if a physician recommends a smart contract system for managing patient data, patients who trust their physicians are more likely to adopt and use it. Increased trust by patients in their physicians leads to better treatment adherence and acceptance of smart technologies [66].

Trust in physicians can mitigate concerns about the safety and efficacy of new technologies. For example, if a physician endorses a smart contract system for managing patient data, patients who trust them are more likely to use it. This perception can reduce anxiety about potential risks, such as data breaches or misuse of personal information. Patients’ acceptance of technological innovation depends on their perceived ability to both use and directly benefit from it, and this is enhanced if their health care provider conveys a positive perception of the technology [67].

An examination of the impact of trust and privacy concerns on technology acceptance in health care found that trust is a direct predictor of patients’ intention to use technology in health care services [68]. A proposed smart contract–based access control framework for securing electronic medical records included mechanisms for user verification and access authorization, which are critical for building trust [69]. The research emphasized that trust in the security and privacy of the system is vital for patient acceptance. When health care providers implement such secure systems, patients’ trust in their providers can translate into a higher intention to use smart contracts.

In primary care, patients rely on their PCP to vet digital tools and to act as a governance steward. These providers can pause or upgrade the contract, hold administrative keys, and know about the policy. We therefore conceptualize provider trust here as trust in the provider’s stewardship of the contract’s governance and upgrade policy, not merely general trust in clinicians.

Hypothesis 3: Greater trust in PCP’s stewardship and vetting of smart contracts is associated with a higher intention to use.

#### Perceived Risks

Perceived risk refers to patients’ beliefs about the potential negative outcomes of using smart contracts, such as financial loss, data breaches, or misuse of personal information. High perceived risk can deter patients from adopting smart contracts, which makes it critical to address and mitigate these concerns. Addressing perceived risks and security concerns related to data access and sharing through robust security measures and clear communication can enhance patient acceptance. It is essential to ensure that data access and sharing are strictly controlled and that patients are informed about how their data are used and protected. Among the key barriers to blockchain-based data management systems, perceived risk was identified as a major challenge [70]. This indicates that higher perceived risk is associated with lower intention to use technologies, such as smart contracts, in health care. A similar study on the impact of trust and privacy concerns on technology acceptance in health care found that perceived privacy risk directly predicted patients’ intention to accept technology in health care services [68].

Perceived risk has been negatively related to the social acceptance of smart health services [71]. Trust in city government and perceived benefits were found to be positively related to social acceptance, while perceived risks and concerns about interventions were negatively related. This suggests that a perception of risk can significantly lower the intention to use smart contracts in health care. Another study that proposed a smart contract–based access control framework for securing electronic medical records emphasized that perceived risks related to data security and privacy were critical factors that affected patient acceptance [69]. Perceived risks related to the reliability, traceability, and security of patient control over medical data also negatively affected patient acceptance [72]. A study exploring how technical, legal, and social factors affected patient privacy evaluations and their acceptance of smart contract applications in health care found that higher perceived risks were associated with lower intention to use smart contracts [51].

Smart contracts are software programs and thus are susceptible to software bugs and cybersecurity risks. Correctness of these programs is fundamentally a software engineering challenge, not just a blockchain issue. High-profile incidents such as the DAO were precipitated by subtle coding flaws; beyond testing, emerging best practices call for formal, machine-readable specifications of required behavior (pre and post conditions, invariants, and temporal properties) and mathematical verification that the code satisfies these properties before deployment [73]. These early technology vulnerabilities shape patients’ security risk perceptions regarding the use of smart contracts in primary care.

Smart contracts introduce irreversibility at the transaction level and shared execution across organizations. In a primary care episode such as a referral, risks include incorrect automated denial or approval, misrouted data, or inability to quickly halt a faulty rule. Patients may specifically worry about key loss or administrator misuse. In our context, perceived risk, therefore, refers to these blockchain-specific risks. Because prior studies consistently suggested that perceived risks related to privacy, security, and data management influence patient acceptance and lower their intention to use smart contracts in health care, we hypothesized the following.

Hypothesis 4: Higher perceived risk from decentralized execution, transaction irreversibility, and governance mismanagement is associated with lower intention to use smart contracts in primary care.

#### Health Care Delivery Type

Patients embedded in traditional primary care practices differ from their typically younger telehealth counterparts in terms of their trust in traditional health care settings and familiarity with conventional care models, which can lead them to perceive higher risks associated with adopting new technologies such as smart contracts [68]. Patients aged 65 years and older have been found to be less likely than patients aged 18-44 years to choose telehealth [74]. Because older patients prefer traditional approaches to primary care more than their younger counterparts, the delivery mode of health care services becomes crucial to patient acceptance of new technologies in health care. Technology-related anxiety and perceived cost have been found to have a negative impact on older users’ behavioral intention to use smart home health care services [75]. Patients in traditional primary care practices are more familiar with conventional care models and may perceive smart contracts as a disruptive technology. This familiarity with traditional care can result in a lower intention to use smart contracts because patients may prefer the known and trusted methods of care delivery [71].

Telehealth patients, on the other hand, are more accustomed to using digital platforms for their health care needs. Telehealth use in primary care practices is associated with lower rates of low-value care services, which suggests that telehealth patients are more open to adopting new technologies that improve care efficiency, such as various digital tools for diagnosis, treatment planning, patient monitoring, and follow-up care [76]. This adaptability and openness to digital health solutions can lead to a higher intention to use smart contracts among telehealth patients compared with traditional patients. Moreover, their prior exposure to digital health technologies can make telehealth patients more comfortable with and accepting of smart contracts, thereby leading to a higher intention to use them compared with patients in traditional primary care settings.

In conclusion, traditional primary care patients are associated with a lower intention to use smart contracts in health care due to their perception of the risks involved in new technologies, their trust in traditional health care settings, and their familiarity with conventional care workflows where face-to-face registration, referrals, and billing are currently manual. In contrast, telehealth patients show a higher intention to use smart contracts. An understanding of these dynamics led to our next hypothesis.

Hypothesis 5: Patients embedded in traditional primary care practices, compared with telehealth-accustomed patients, are associated with a lower intention to use smart contracts that automate primary care workflows.

**Figure 1.**
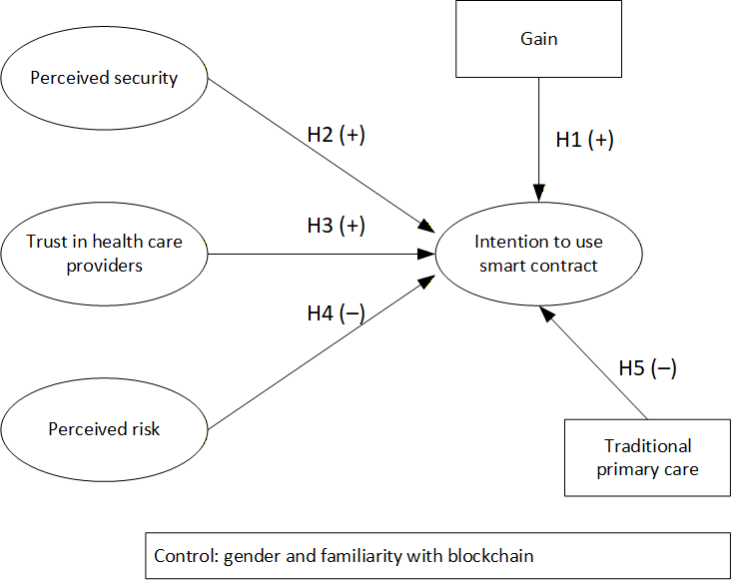
Conceptual model.

Figure 1 shows the conceptual model, which incorporates all 5 hypotheses.

## Methods

### Data Collection and Summary

**Figure 2.**
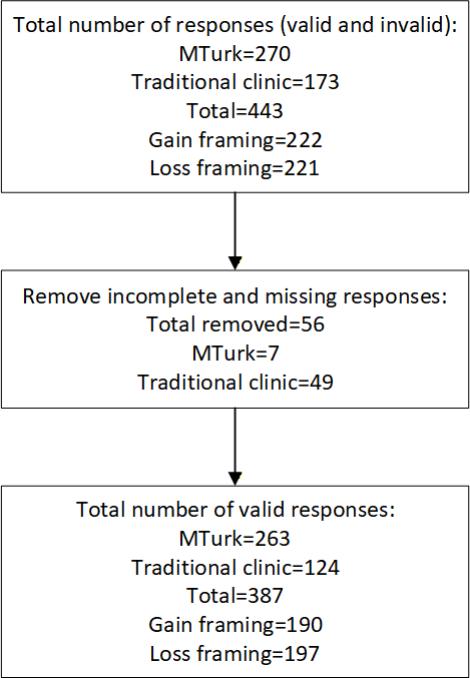
Data collection and cleaning process.

The total number of respondents, including complete and incomplete responses, was 443. Figure 2 summarizes the data collection and cleaning process. Of the 443 responses, 270 were collected via Amazon MTurk and 173 were collected via the traditional clinic. The number of gain- vs loss-framed assignments was balanced at 222 and 221, respectively, due to random assignments at 50%. However, 56 respondents had incomplete responses, which resulted in the removal of these records from the final study analysis. Of the 56 invalid responses, 6 were from MTurk and 49 were from traditional clinic respondents. The traditional clinic has 30 patient visits flow per day. We ran the study for 2 weeks, which resulted in collecting data from 173 patients (124 valid responses) out of approximately 300 patients.

**Figure 3.**
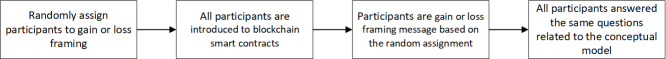
Study design.

A total of 387 valid and complete responses were used in the study analysis from participants recruited through both channels, MTurk and the traditional clinic. A total of 124 responses were from the traditional patients, and 263 responses were from MTurk participants. The clinic patients represented patients embedded within traditional primary care practice as discussed in hypothesis 5. Of the 387 participants, 194 were female and 193 were male. Participants were randomly assigned to 1 of 2 scenarios (gain framed and loss framed) in which they were introduced to blockchain smart contracts and how they can be used in health care. The first paragraph of the gain-loss-framed message, which introduced a simplified explanation of blockchain and smart contracts, was identical for both groups. The second paragraph explained how smart contracts could be used in health care. Both scenarios had the same information and length of paragraphs. However, the gain-framed group read a paragraph with a positive sentiment, while the loss-framed group read a paragraph with a negative sentiment. Figure 3 shows the study design. Of the 387 completed responses, 197 participants were randomly assigned to the loss-framed condition and 190 participants were assigned to the gain-framed condition. Textbox 1 shows the paragraphs that were presented to the participants.

Uncited Textbox 1. **Blockchain and smart contracts farming.**

Read the following text carefully before starting the survey.

**What is a blockchain smart contract, and how can it be used in health care?**

- A database is a tool for collecting and organizing information. Databases are used in your health care provider’s office to collect, track, and store information on your personal information, insurance, diagnosis, treatment, prescription drugs, laboratory tests, and hospitalization. Blockchain technology is an advanced database that allows transparent information sharing within a business network. Smart contracts are programs stored on a blockchain that run when predetermined conditions are met. They are typically used to automate an agreement without a third party so that all participants can immediately get an outcome.

**Gain**

- In health care, smart contracts can be used in several ways: faster, more convenient check-in and check-out at the doctor’s office, urgent care, or in the emergency room. With the smart contract, providing your medical history is much easier and less repetitive. The smart contracts allow participating doctors convenient, easy, and immediate access to your health information. Smart contracts can enable faster care decisions, thus providing greater opportunities for care coordination. For example, patients could get instant approvals for MRIs, medication, and medical procedures when necessary. Your health care provider could get paid immediately after your medical visit. That data on the smart contracts cannot be deleted or edited; corrections require adding data without deleting the old record. Smart contracts allow for efficient tracking of errors and, therefore, improve accountability.

**Loss**

- In health care, when smart contracts are not in use, checking in and out of the doctor’s office, urgent care, or the emergency room takes a very long time. Unfortunately, you waste time repeating your medical and family history without smart contracts. Without smart contracts, your doctors have limited access to your health information, so decision-making is slower, and it limits their ability to provide improved care coordination. For example, approvals for MRIs, medication, and medical procedures could take several days or weeks. Your health care provider will wait 30-60 days to be paid for your medical visit. Without smart contracts, the data in your electronic medical record can be deleted or edited, without your knowledge, and you have no way of tracking errors or holding responsible parties accountable.

All questions in the survey were the same for all participants. After reading the introductory text (provided in Textbox 1), the participants answered questions related to their intention to use smart contracts in health care, as well as perception-based questions related to risk, security, and trust in health care providers. The table in Multimedia Appendix 1 shows the latent variable questions in the conceptual model. All latent variables used a 5-point Likert scale (strongly disagree to strongly agree). Each latent variable had 3 items. Participants were also asked to provide their gender, year of birth, race, number of health conditions, and type of insurance.

### Statistical Analysis

We used IBM SPSS Statistics to manage and clean the data and IBM SPSS Amos (version 29) for the confirmatory factor analysis to assess the measurement model and structural equation modeling (SEM) to test the conceptual model. The 5 hypotheses included in the conceptual model were as follows: gain-loss-framed messaging positively impacts the intention to use smart contracts, the perceived security of smart contracts and trust in physicians are associated with a higher intention to use smart contracts, perceived risk is associated with a lower intention to use smart contracts, and patients in traditional health care settings are associated with lower behavioral intention to use smart contracts compared with their telehealth counterparts.

### Ethical Considerations

**Figure 4.**
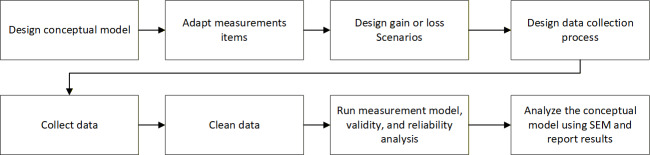
Study procedure. SEM: structural equation modeling.

We first applied to the institutional review board for approval to collect data. The inclusion criteria included participants of all genders aged 18 years and older. Individuals who were unable to read or comprehend English were excluded from the study. The application included a proposal to collect data through 2 channels: Amazon Mechanical Turk (MTurk) and a primary care clinic in upstate New York. Participants in the traditional primary care setting were recruited during the registration process when they arrived for an appointment. We received exempt approval for the study (reference no 23‐328). Participants from both channels were informed that the survey was voluntary and anonymous, and that they could stop the survey at any time. A modest compensation was given to the participants. Figure 4 shows the study procedure.

## Results

### Measurement Model Assessment

Table 1 shows the confirmatory factor analysis results of the measurement model. All factor loadings for the individual items were high and significant. The factor loadings ranged from 0.814 to 0.895. The model fit indices were in line with a good model fit. For example, both the comparative fit index and the Tucker-Lewis index were well above the cutoff for a good fit criterion [77] at 0.975 and 0.965, respectively.

**Table 1.** Measurement model.

Latent variable and item	Loadings	AVE^a^	Construct reliability	Variable inflation factor (VIF)
Intention to use (INT) smart contracts (dependent variable)		0.74	0.895	—^b^
INT_1	0.891			
INT_2	0.814
INT_3	0.873
Perceived security (PS)		0.765	0.907	2.728
PS_1	0.895			
PS_2	0.839
PS_3	0.889
Trust in health care providers (TRP)	0.728	0.889	2.561
TRP_1	0.869			
TRP_2	0.822
TRP_3	0.868
Perceived risk (PR)	0.713	0.882	1.316
PR_1	0.863			
PR_2	0.818
PR_3	0.852
Model fit				
Comparative fit index (CFI)	0.975	—	—	—
Tucker-Lewis index (TLI)	0.965	—	—	—
Root mean square error of approximation (RMSEA)	0.07	—	—	—

a AVE: average variance extracted.

b Not applicable.

### Reliability and Validity

As shown in Table 1, the construct reliability showed strong reliability for all latent variables, with scores ranging from 0.882 to 0.907. The constructs also met the validity criteria, which were assessed using average variance extracted (AVE). The AVE scores for all latent variables were well above the 0.5 cutoff, with the lowest score being 0.713. In addition, the AVE scores exceeded the squared multiple correlation scores with other variables. Finally, the variance inflation factor scores for all independent latent variables were well below the cutoff of 5, indicating that the model did not suffer from multicollinearity.

### SEM Results

The conceptual model was tested using SEM. The model fit indices for the conceptual model were as follows: comparative fit index=0.933, Tucker-Lewis index=0.918, and root mean square error of approximation=0.085. The root mean square error of approximation of 0.085 is slightly above ideal cutoff but still within the acceptable range of 0.08 and 0.1 [78]. In addition, the SEM results of the conceptualized model are shown in Table 2. Paths in the SEM represent associations among latent variables in cross-sectional survey data, and we refrain from making causal claims. Any directional language in figures follows conceptual ordering, not identification of causal effects. All of our hypotheses were supported except for the gain-loss effect. The results showed that message framing had no significant influence on the participants’ intentions to use smart contracts, at least in this context. In other words, gain- or loss-framed messages did not seem to influence individual intentions to use smart contracts. This finding was contrary to the hypothesis developed in the theoretical background. In the “Discussion” section, we attempt to explain why this result was observed.

**Table 2.** The conceptual model results.

Variable	Standardized estimate	*P* value
Perceived security	0.50	<.001
Trust in health care providers	0.43	<.001
Perceived risk	−0.07	.048
Gain scenario	0.01	.85
Male	−0.07	.02
Clinic	−0.14	.001
Familiarity with blockchain	−0.08	.07

Hypothesis 2 proposed that perceived security is associated with a higher intention to use smart contracts. As discussed in the background section, increased security is one of the characteristics and advantages of blockchain networks. This hypothesis was supported by the results, as shown in Table 2. The perceived security estimate was positive and significant, with an estimate of 0.504 and a *P* value of <.001. In fact, the perceived security standardized estimate of 0.504 was the highest among all of the influencing variables in the model. Therefore, perceived security seems to have the largest influence on the intention to use smart contracts. This result has implications for health care providers to emphasize the security benefits of blockchain and smart contracts to increase patients’ adoption. It is important to note that the sample represents a single traditional clinic and online participants.

Hypothesis 3 stated that trust in providers is associated with a higher intention to use smart contracts. The results showed that this relationship was positive (0.433) and significant (*P*<.001), thereby providing support for this hypothesis. Thus, individuals reporting greater trust in their PCP reported higher intentions to adopt smart contracts. This is a significant finding because the COVID-19 era revealed a lack of trust between individuals and the health care industry. It is important to work on building stronger trust between providers and patients because trust has been shown to have a direct influence on individuals’ decisions to adopt instructions, technologies, or medications provided by health care providers.

Hypothesis 4 emphasized that perceived risk is associated with a lower intention to use smart contracts. The hypothesis was supported with a significant negative estimate of −0.071. However, while the influence was significant, its magnitude was much smaller than perceived security and trust in health care providers. Therefore, health care providers should surface concrete assurances, such as visible audit trails for cross-party actions and clear, governed pause or upgrade controls, while building trust, thereby reducing the negative impact of perceived risk.

The final hypothesis examined whether patients in traditional primary care settings would be associated with a lower intention to use smart contracts. The results supported this hypothesis. In addition, an interesting result from one of the control variables was that male participants, compared with female participants, were associated with a lower intention to use smart contracts.

## Discussion

### Principal Findings

The findings of this study indicate that the disruptive technology of blockchain smart contracts can be successfully implemented in primary care. The results aligned with the hypotheses, which predicted that patients’ behavioral intentions to use smart contracts in health care would be influenced by their trust in their health care providers and their perceptions of the security and risks involved in smart contracts [73,79]. The study extends previous research on technology acceptance in health care by specifically focusing on the implementation of smart contracts in primary care, thereby making it one of the first papers to address this topic.

The 2 scenarios presented in this study emphasized both the benefits of using smart contracts and the costs of not using them. Interestingly, neither framing significantly influenced participants’ intentions to use smart contracts. This finding contrasts with the general effectiveness of message framing in health care when patients are uncertain about the impact of a situation [80]. Patients’ increasing familiarity with disruptive technologies, such as artificial intelligence [81,82] and mobile apps [83], could have resulted in the lack of a framing effect. That is, their interaction with other disruptive technologies could have mitigated their uncertainty about smart contracts. Future research can examine this finding to understand how familiarity with disruptive technology shapes patient attitudes toward technology adoption. Our vignettes described generic efficiency gains and delays and had low stimulus specificity. This may have encouraged respondents to answer as if they were responding to digital modernization in general, thereby diluting classic gain-loss effects. Additionally, in our SEM, the standardized paths for perceived security (*β*=.50) and trust in providers (*β*=.43) dominate the framing path, plausibly swamping small framing effects. Moreover, framing effects in health decisions frequently depend on moderators such as familiarity, involvement, risk attitude, and numeracy. Blockchain familiarity and care setting, as reflected in our study design, may have been insufficient to surface framing effects. As health care providers consider implementation strategies, they should focus on educating patients about the advantages of smart contracts for care delivery rather than relying solely on message framing to increase adoption.

Consistent with our findings, perceived security is a salient antecedent of intention, especially given recent data breaches and ransomware attacks that have resulted in the loss of millions of patients’ protected health information to bad actors. Effective designs should communicate how safety is achieved (eg, auditable histories and governed responsiveness) rather than restating generic benefits. As blockchain technology gains mainstream attention in the investment markets through applications such as Bitcoin, health care providers should capitalize on this awareness to demonstrate how smart contracts can protect patient data and improve health care processes.

Smart contracts are executed on decentralized platforms hosted by unknown third parties. In many industries, this feature is seen as valuable; however, it could be seen as a barrier to adoption in health care. Patient data contains highly sensitive information, and if patients perceive that their data may be intentionally divulged or compromised by unknown third parties, their intention to use smart contracts may be impacted. As hypothesized, this study found that having a primary care physician as an intermediary in this “trust-less” [84] security apparatus can improve the likelihood of smart contract use. This finding is supported by previous research showing that patients’ behavioral intentions to use mobile technology are impacted by their trust in their physicians [66]. Health care professionals should capitalize on the patient-physician relationship to promote the adoption of smart contracts in primary care by carefully highlighting the physician’s role in overseeing and supporting the implementation process. Specifically, trust in the provider may serve as a bridge facilitating smart contract adoption. In the future, primary care physicians could monetize this intermediary role to supplement their practice income. For example, physicians could be compensated on a per member per month basis, similar to value-based contracts, for each patient who opts to use the smart contract.

Risk is often a significantly restraining construct for technology adoption [85]. Notably, older adults generally perceive a higher level of risk when adopting technology in health care [86]. In alignment with prior studies, participants in this study were also influenced by their perception of risk. Interestingly, although risk had a negative impact on patients’ intention to use smart contracts, the magnitude was small. This result could be attributed to smart contract technology being implemented in a familiar environment and endorsed by a trusted physician. When implementing smart contracts in traditional or nontraditional primary care settings, providers should leverage the patient-physician relationship to help mitigate perceived risks. Another factor impacting the magnitude of the risk effect may be patients’ perception of the security of smart contracts. While potential risks are high, security could be seen as a factor that mitigates risk. The lower magnitude of risk should not be considered negative; rather, it should be used to guide implementation strategies. For example, known risks can be discussed with patients during routine visits to help reduce their impact.

Compared with online participants, the individuals in a primary care office were generally older and had multiple chronic comorbidities; they were also less likely to intend to use smart contracts than the online participants. Additionally, an interesting finding from the control variables revealed that males were associated with a lower intention to use smart contracts compared with females. Prior research found comparable results and showed that gender, age, and complex health conditions negatively impacted technology adoption [87-89]. Health care providers and technology developers should customize implementation strategies to consider patient demographics and provide support and education for older patients, especially those with complex health conditions.

Smart contracts have improved workflows and patient outcomes in secondary and tertiary care. This study can help health care professionals and technology developers understand the factors that affect adoption in the primary care setting. The successful implementation of this disruptive technology depends on an in-depth understanding of patients’ trust in physicians, their perceptions of the security and risks involved in smart contracts, and patient characteristics, so that health care providers can design adoption initiatives. This study’s findings contribute to the extant literature on blockchain applications in health care and offer practical guidance for primary care providers looking to leverage smart contracts to improve patient outcomes and streamline health care processes.

Because smart contracts encode business, and at times legal logic across organizations, expressing the intended behavior as formal, machine-readable specifications enables mechanized checks that critical properties hold (eg, no payment without a valid referral and authorization; emergency pause overrides cannot be bypassed). Techniques such as model checking, theorem proving, symbolic execution, and property-based testing can then mathematically verify that implementations meet these specifications before deployment. This is especially salient in health care, where errors can carry serious consequences. Our results highlight perceived security as a key antecedent; communicating that primary care contracts are formally specified and verified provides a concrete assurance basis beyond narrative claims of “security,” and aligns assurance evidence with patient expectations.

### Conclusions

Our study addresses a clear gap in the healthcare blockchain literature by examining patient intentions to use smart-contract workflows in primary care, where day-to-day activities like referrals, prior authorization, and co-payment settlement require multi-party coordination and credible assurance mechanisms. Using a cross-sectional SEM, we find that perceived security and trust in provider stewardship are positively associated with intention to use, while perceived risk shows a negative but comparatively small association. In contrast, our message-framing manipulation (gain vs. loss) was not associated with patient intentions to use.

Altogether, our results suggest that adoption in primary care hinges less on promotional framing and more on visible assurances and governance. Effective designs should show how safety is achieved, through tamper-evident, cross-party audit trails and governed responsiveness, rather than re-stating generic benefits. Trust in the primary care physician appears to function as a bridge for adoption in a “trust-minimized” infrastructure. The clinician acts as a steward of governance and change management can strengthen patients’ willingness to engage with smart-contract-mediated processes. We also observe an important heterogeneity. In-clinic participants, older patients with more comorbidities, reported lower intentions than online participants. Additionally, men reported lower intentions than women, underscoring the need to tailor outreach and support for specific demographic groups. Beyond user-facing communication, the findings highlight that perceived security can be bolstered by engineering evidence, not messaging alone. Introducing and communicating formal, machine-readable specifications of contract behavior paired with verification can provide mechanical assurance that critical properties hold before deployment. In primary care, succinct assurance disclosures may credibly raise perceived security and, in turn, intention to use.

In summary, this study advances understanding of patient acceptance of smart contracts in primary care and offers practical guidance. These include providing concrete assurances, leveraging provider stewardship, addressing risk through dialogue and design, and backing claims with verification evidence. Aligning these elements can help primary care organizations realize the benefits of programmable, auditable, and governable coordination while maintaining patient trust.

### Limitations and Future Work

While this study provides valuable insights into patient perspectives on the use of smart contracts in primary health care, there are limitations that need to be acknowledged to contextualize the findings and guide future research. First, we were limited by our dataset because self-reported surveys tend to be susceptible to biases, such as social desirability or recall errors. Because the design is cross-sectional and relies on intentions, endogeneity cannot be ruled out, and findings have been interpreted as conditional associations. Although efforts were made to explain the concept clearly, varying levels of digital literacy among respondents could have influenced their responses. In addition, the collection of actual behavioral data is very challenging because the implementation of smart contracts in health care is in its infancy stages. Another limitation is that the data were collected from one primary care provider in upstate New York. Future research could benefit from soliciting responses from patients in various locations and states. In addition, the ratio between MTurk and clinic patients was skewed toward MTurk participants, with 263 out of the 387 participants. This might weaken the external validity of the study. As mentioned in the data collection section, data collection from patients in actual clinics was very challenging. Most of the invalid and incomplete responses were from participants at the clinic.

While most of the hypothesized relationships were supported, including the strong positive effects of perceived security and trust in health care providers, the gain-loss framing effect was not supported. This may suggest that patients do not yet have sufficient context or familiarity with smart contracts to meaningfully differentiate outcomes framed as gains vs losses. Future research needs to consider alternative framing strategies or deeper educational interventions before measurement.

Although the subgroup differences in the acceptance of smart contracts were significant, the cross-sectional design limited causal interpretations. Longitudinal studies could help determine whether these differences persist over time or shift with greater exposure to smart contract technologies. While diverse in terms of certain demographic and usage profiles, the current sample may not fully represent populations with limited access to digital health care, older adults, or individuals with low health literacy. Future studies need to use mixed method approaches, expand sampling across health care settings and patient types, and incorporate behavioral data from actual smart contract usage to enhance the validity and applicability of the findings.

It would be worthwhile to design studies in which the scenarios differ in the type of information they emphasize regarding the benefits of blockchain and smart contracts. For instance, one scenario could highlight accountability, another could focus on security, and another on transparency. This approach would allow us to examine which aspects of blockchain and smart contracts are most influential from the patients’ perspective. Future work should also test whether assurance disclosures increase perceived security and intention compared with generic security claims and should report verification artifacts alongside clinical outcomes in field deployments.

Future studies could examine how perceptions of different security dimensions (eg, ledger robustness, smart contract code safety, and data governance) influence the adoption of smart contracts. In addition, future studies should incorporate measures of digital literacy and health literacy to better understand how varying literacy levels influence perceptions of blockchain smart contracts.

Future research could also explore several critical areas to advance the practical and systemic application of smart contracts in the health care domain. One such research direction involves evaluating the real-world implementation of smart contracts from patients’ perspectives. While this study focused on perceived acceptance, future longitudinal studies could assess patient satisfaction, trust, usability, and health outcomes in settings where smart contracts are actively used for managing appointments, consent, and billing workflows.

Blockchain technologies automate and streamline administrative workflows, thereby potentially reducing burnout. Therefore, another vital research path involves understanding how provider perceptions evolve with the actual use or mandated adoption of smart contracts. Future studies could investigate changes in trust, workload, documentation efficiency, and administrative burden among clinicians and health care staff. Smart contracts can also automate repetitive tasks for physicians, such as managing referral chains, authorization checks, and follow-up reminders. Future work could prototype and evaluate smart contract–based systems that integrate with EHR platforms to automate these functions and analyze their impacts on provider time management and care coordination.

A transformative area of inquiry is how smart contracts might reshape payment models in primary care. Specifically, the shift toward value-based payment models, such as the patient-centered medical home program, could benefit from smart contracts that automatically validate and trigger payments upon meeting predefined quality metrics. Research could explore frameworks for encoding such contracts and pilot studies to measure financial and clinical outcomes.

Smart contracts also have the potential to transform claims processing in health insurance by enforcing logic-based rules that match submission formats, such as the 5010 standard for HIPAA-compliant transactions. Research could prototype and evaluate such systems within primary health care networks and determine their interoperability with clearinghouses and insurers. Furthermore, smart contracts can help address semantic and data consistency issues in Fast Healthcare Interoperability Resources (FHIR) and EHR integration by ensuring consensus-driven logic for health data exchange. Investigating how FHIR-compatible smart contract programs could reduce discrepancies and improve clinical data quality will be essential.

Policymakers also have a pivotal role to play. Future studies should analyze how federal and state policy frameworks can be adapted or developed to ensure cybersecurity, patient data privacy, and the ethical deployment of smart contracts in the Medicare and Medicaid programs. These studies should also explore the patient-centric payment models and reimbursement logic encoded in smart contracts that promote transparency and equity. Future research should also examine how smart contracts can support the population’s health and public health initiatives, such as vaccination tracking, chronic disease management, and outbreak reporting. These programs often suffer from data silos and inefficiencies, which blockchain-based automation could alleviate. A sociotechnical analysis of deploying such solutions at scale, especially in underresourced or rural settings, would contribute significantly to both the blockchain and public health literature.

We believe future research can bridge the gap between the conceptual promise and the technical, organizational, and regulatory realities of smart contract use in primary health care. By taking a multidisciplinary approach, future work can support the development of secure, efficient, and patient-centered digital health ecosystems.

## Supplementary material

10.2196/82237 Multimedia Appendix 1 Measurement items.

## Abbreviations

AVE: average variance extracted
DAO: decentralized autonomous organization
EHR: electronic health record
FHIR: Fast Healthcare Interoperability Resources
HIPAA: Health Insurance Portability and Accountability Act
MTurk: Amazon Mechanical Turk
PCP: primary care physician
QoS: quality of service
SEM: structural equation modeling
