# Supplementary material for: Acceptance of Smart Contracts in Patients Receiving Primary Care: Exploratory Study
Source: JMIR Form Res. 2026 Mar 12;10:e82237. doi: 10.2196/82237 (PMC12990173; doi:10.2196/82237)
Supplement: Multimedia Appendix 1 [file formative-v10-e82237-s001.docx]

| **Variable** | **Code** | **Items** | **Adapted from** |
| --- | --- | --- | --- |
| Intention to use smart contracts (dependent variable) | INT_1 | I am willing to use smart contracts in a healthcare context. | [1] |
|  | INT_2 | I will probably use smart contracts in a healthcare context. |  |
|  | INT_3 | I am likely to use smart contracts in a healthcare context. |  |
| Trust in Healthcare Providers | I believe that healthcare providers…. | | [2] |
|  | TRP_1 | are credible. |  |
|  | TRP_2 | are trustworthy. |  |
|  | TRP_3 | adhere to a set of principles (e.g., honesty and promise keeping) that I (the patient) find acceptable. |  |
| Perceived Risk | PR_1 | The decision to use smart contracts in healthcare is risky | [4] |
|  | PR_2 | I believe that using smart contracts in healthcare can be harmful |  |
|  | PR_3 | Using smart contracts in healthcare involves more risk than using traditional healthcare systems |  |
| Perceived security | PS_1 | My personal health information would be securely managed in blockchain smart contracts | [5] |
|  | PS_2 | Blockchain smart contracts are safe for my personal health information |  |
|  | PS_3 | Blockchain smart contracts are secure for my personal health information |  |
| Control Variables | | | |
| Familiarity with blockchain |  | Were you familiar with blockchain before starting this survey? (Yes/No) |  |
| Familiarity with smart contracts |  | Were you familiar with smart contracts in blockchain before starting this survey? (Yes/No) |  |
| Age |  | When were you born? (year) |  |
| Gender |  | Male/Female/Other |  |
| Race |  | a) White not Hispanic e) Asian  b) Black or African American f) Native Hawaiian and other Pacific  c) Hispanic or Latino g) Other  d) American Indian or Alaska Native |  |
| Income |  | What is your income? (range) |  |
| Education |  | What is your level of education? |  |
| Health Status |  | Do you have any chronic medical conditions? (enter the number) |  |
| Insurance |  | What type of health insurance do you have? **Medicare or Medicare Advantage** (Senior or Disabled), **Medicaid** (Low or No-income), **Commerical** (PPO – I can go to any Dr,) **Managed Care** (HMO – I have to go to specific doctors in my network), **Tricare**, **CHAMPVA**, **No Health Insurance** (self-pay) |  |
|  |  |  |  |

**References**

1. Malhotra, N.K., S.S. Kim, and J. Agarwal, *Internet users' information privacy concerns (IUIPC): The construct, the scale, and a causal model.* Information systems research, 2004. **15**(4): p. 336-355.

2. Abdelhamid, M., *Greater Patient Health Information Control to Improve the Sustainability of Health Information Exchanges.* Journal of biomedical informatics, 2018.

3. Dinev, T. and P. Hart, *An extended privacy calculus model for e-commerce transactions.* Information Systems Research, 2006. **17**(1): p. 61-80.

4. Harris, M.A., R. Brookshire, and A.G. Chin, *Identifying factors influencing consumers’ intent to install mobile applications.* International Journal of Information Management, 2016. **36**(3): p. 441-450.

5. Hartono, E., et al., *Measuring perceived security in B2C electronic commerce website usage: A respecification and validation.* Decision support systems, 2014. **62**: p. 11-21.
